# Hospital Reform

**Published:** 1897-11-27

**Authors:** 


### EDITOR'S LETTER-BOX.

[Our Correspondents are reminded that prolixity is a great bar to publication, and that brevity of style and conciseness of statement greatly facilitate early insertion.]

### HOSPITAL REFORM.

Mr. Garrett Horder, Hon. Secretary Hospital Reform Association, writes: I shall be glad if you will allow me to make a few observations on the article you published last week. In the first place, instead of saying that hospitals are charities, you should have said hospitals ought to be charities. There are, as you are aware, many hospitals in London where the patients are called on to pay sums varying from 3d. to 2s. 6d. for advice and medicine. I may remind you that even at Guy's Hospital people are expected to pay threepence for every bottle of medicine had in the out-patients' rooms, and that in the dental department of that hospital there is a regular scale of charges for the various operations on the teeth of patients. If you refer to our report on the special hospitals you will find that thirty-two of them receive payments from their patients, and that at eighteen of them the amount received in this way is larger than the amount received from their subscribers. It cannot be doubted that these patients, who are called upon to pay sums, however small, for medicines, are led to believe that they pay full value for them. It is also manifest that this system of patient payment attracts a class of persons who do not belong to the class of necessitous poor. If people find that they can, by paying small sums, receive treatment at hospitals, it follows that they will not make provision for sickness when in health. Such a system is therefore a great bar to the promotion of thrifty habits amongst our working-class population. It is perfectly clear that so long as our hospitals open their doors so freely to all comers, people who do not suffer from ailments requiring the special attention of skilled physicians and surgeons will resort to them. It is also perfectly clear that hospitals will continue to find it difficult to meet the demands of this large class of persons. The expenditure necessary for more capacious waiting-rooms, consulting-rooms, &c., must have the effect of crippling the income of hospitals, and must at times lead to the curtailment of the number of in-patients. I am afraid that the proposals you advocate would be likely to further increase the abuse of the out-patient department.

However stringent the regulations made people would naturally conclude that admission to hospitals would be gained by producing the stamp book; and, moreover, it would be found that the hospital authorities would be much more likely to give precedence to such persons as brought such a book. Like subscribers' letters these stamp books would be a great source of abuse, and it is more than probable that the really deserving poor would suffer neglect. Might I suggest that you should take up the question of provident dispensaries, and see if some plan of co-operation could not be devised whereby such institutions could have the advantage of drafting off special and serious cases to the hospitals. The right idea is to utilise the out-patient department as a consultative one. If general practitioners found that hospitals only received such cases as they (the general practitioners) thought required and deserved hospital treatment, not only would the hospitals cease to be abused, but they would become the means of bring about a more cordial relationship between the consultants and the main body of the profession. Men in general practice, when they discovered that the hospital staffs were anxious and willing to give their advice in the treatment of difficult or obscure cases, would not for one moment hesitate to avail themselves of that advice. Under such a system the patients would benefit, the hospitals would cease to be abused, and the profession would have no grievances to complain of.

*** Mr. Garrett Horder seems not to recognise that whatever is done the first essential is what we have advised, viz., an inquiry officer to investigate the cases. If Mr. Garrett Horder proposes that fit cases shall not receive hospital treatment unless recommended by a medical man we feel sure that he will not receive the support of hospital governors, while if he proposes that unfit cases shall be admitted to treatment on presenting such a recommendation we fail to see where the reform comes in.—Ed. *T.H.*

